# Diagnostic and Predictive Biomarkers in Lung Cancer

**DOI:** 10.3390/cancers13112577

**Published:** 2021-05-25

**Authors:** Caterina Fumagalli, Massimo Barberis

**Affiliations:** Division of Pathology, IEO, European Institute of Oncology, IRCCS, 20141 Milano, Italy

Lung cancer is the most frequent cause of cancer-related mortality worldwide. An early diagnosis, the identification of conditions predisposing to lung cancer, and the definition of the tumor genetic profile are crucial steps to improved patient outcomes. In the present special issue, “Diagnostic and Predictive Biomarkers in Lung Cancer”, these important topics are covered across fourteen peer-reviewed papers produced by recognized experts on the subject.

We have the pleasure of presenting a multicenter European study coordinated by Giulia Veronesi: “Lung Cancer Screening with Low-Dose Computed Tomography” [1]. The authors stated that the implementation of such screening is urgently needed in Europe in order to diagnose lung cancer at the early stages and reduce mortality rates. The group propose shared recommendations such as the implementation of a risk-prediction model to select high-risk populations or the management of lung nodules by utilizing volume and growth-rate data with more stringent cut-offs.

On a different topic, specific conditions such as idiopathic pulmonary fibrosis (IPF), a severe progressive interstitial lung disease, may be associated with lung cancer development. Indeed, up to 15% of IPF patients develop lung cancer within five years. Miriana D’Alessandro and colleagues [2,3] completed two studies focused on the identification of biomarkers linked to prognosis and therapy response in patients affected by idiopathic pulmonary fibrosis. In detail, the authors reported the utility of the most common, routinely available oncomarkers (namely NSE, CEA, Ca19.9, and Ca125) in the evaluation of IPF patients’ clinical courses [2]. They also identified the serum concentration of Krebs von den Lungen-6 (KL-6) as a helpful biomarker to be monitored in IPF patients during antifibrotic therapy, as stable levels were associated with stabilization of lung function parameters. Moreover, a KL-6 serum level increase was observed in IPF patients who developed lung cancer and was associated with poor prognosis [3]. Both these studies add valuable insight into the clinical management and follow up of IPF patients.

Furthermore, we should consider that 30% of patients are diagnosed at an early stage, and have resectable lung cancer. In these cases, a surgical approach of lobectomy/pneumonectomy could be applied. The interesting study by Marjanski and colleagues [4] evaluated a useful “low-tech” exercise of a 6 min walking test (6MWT) for prediction of complications and overall survival after pneumonectomy. As reported, patients with poor results in the 6MWT the day preceding the surgery were at high risk of postoperative cardiac complications and had poor overall survival.

Although great efforts have been made to improve screening and early tumor detection, up to 70% of patients are diagnosed at advanced stages and have unresectable disease. In advanced and metastatic contexts, the genomic portrait of the tumor takes on a pivotal role in therapy selection. Indeed, the detection of oncogene driver mutations in non-small-cell lung cancer (NSCLC) can be used to identify patients with potential survival benefit from targeted therapies, and PD-L1-positive staining in oncogenic-not-addicted tumors can help to identify patients as candidates for immunotherapy. However, the potential therapeutic options have moved beyond the standard-of-care *EGFR*/*BRAF*/*ALK*/*ROS1* and PD-L1 evaluations. In this evolving context, where the number of actionable genetic alterations to be tested is constantly increasing but the tumor tissue may be scarce, a comprehensive genomic profile determined using next-generation sequencing (NGS) panels could be the most effective compromise. How to implement tumor genomic profiling in routine clinical settings is still debated; the Italian Association of Thoracic Oncology (AIOT) produced a position paper summarizing the results of a discussion from a Precision Medicine panel meeting on the challenges facing the introduction of a comprehensive genomic profile (CGP) into daily clinical practice [5]. The study conducted by De Luca and colleagues [6] presents a narrow RNA fusion panel used to investigate gene fusions and splicing events that could be successfully implemented in a routine diagnostic setting. Antonio Passaro and coworkers [7] discussed the characterization of concurrent driver alterations in NSCLC, which is helpful for personalized treatment selection and detection of resistance. Moreover, NGS analysis could increase understanding of the biology of tumor development and progression. As reported by Centoze [8], the four World Health Organization (WHO) classes of lung neuroendocrine neoplasms are characterized by specific molecular alterations that could predict the clinical course and guide therapy selection. Nevertheless, performing a CGP of NSCLCs may produce a plethora of molecular data that are tricky to manage and difficult to translate into clinical indications. To this end, we report the experience of a multidisciplinary molecular tumor board (MTB) as a valuable tool to support patients’ physicians to apply precision medicine strategies [9].

The field of biomarkers in NSCLC is protean: different targets, different methods, and different clinical contexts. Besides tissue-based markers, ctDNA, proteomic, epigenomic, and metabolomic markers have recently enlarged this constantly evolving panorama. In particular, the liquid biopsy, based on the detection of tumor-related biomarkers in body fluids such as peripheral blood, has emerged as a very promising and noninvasive diagnostic tool that can capture spatial and temporal intratumoral heterogeneity. Florian Janke and coworkers [10] described how liquid biomarkers can be tested to assess responses to therapy in advanced NSCLCs. The use of liquid biomarker panels might help to adjust treatment earlier than currently possible using radiographic tumor assessments, and thus avoid or shorten the side effects and adverse events associated with ineffective treatment. Paul Hofman [11] elegantly discussed the impact of next-generation sequencing in its application to liquid biopsies in treatment-naïve NSCLC patients. The author thoroughly investigated different issues regarding the implementation of liquid biopsy as a complementary or alternative option to tissue biopsy for treatment-naïve advanced NSCLC patients. Although this is a very exciting new area, NGS performed on circulating nucleic acids at the point of diagnosis may have limitations, and we should be aware that negative results could be due to the lower level of performance of molecular biology analyses performed via liquid biopsy compared with tissue biopsy.

The main goal in the clinical management of lung cancer patients is to schedule the most effective course of treatment for the individual patient, as different drugs are available and may be used in combination or sequentially to overcome resistance mechanisms. Towards this aim, different elements should be considered, including tumor-related and tumor-independent factors. Indeed, sex-based differences could also affect disease clinical courses. Pérez-Díez and coworkers [12] compared transcriptomic data obtained from lung adenocarcinomas that developed in male and female patients and highlighted different functional signatures that could influence tumor progression and response to therapy. Regarding the benefits of immunotherapy, nontumor factors such as the host immune system or microbiota could play a central role. To date, PD-L1 immunostaining alone may not be sufficient, but new biomarkers are emerging, such as tumor mutational burden (TMB) and tumor-infiltrating lymphocytes (TILs). On this topic, Sesma and colleagues [13] provide a review, identifying tumor mutational burden (TMB) and blood T-cell receptor TCRβ (TCRB) as promising predictive biomarkers for immunotherapy response.

Lastly, the evolving molecular landscape of neuroendocrine tumors of the lung, mainly small-cell lung cancers (SCLC) and large-cell neuroendocrine carcinomas (LCNEC) deserves a special mention for its therapeutic implications. Different chemotherapy schemes applied according to molecular biomarkers could offer new opportunities to patients with LCNEC. The study of Carazo and colleagues [14] reports an innovative genome-wide loss-of-function screen that may be useful for identifying biomarkers predictive of therapy response, especially for tumors with scarce therapeutic options. Applying this tool, the authors identified CREBBP-defective SCLC to be vulnerable to polo-like kinase 1 (PLK1) inhibition. 

In conclusion, precision cancer medicine is merging the tumor genomic alteration landscape with available therapies, aiming to improve patient outcomes. The great challenge for the pathologist is to guide treatment accurately, which requires going beyond diagnosis and tumor classification to give clinically useful diagnostic, prognostic, and predictive parameters within a timeframe consistent with clinical needs.

This special issue of *Cancers* highlights the current state-of-the-art technology in diagnostic and predictive assays used for lung cancer treatment, with special emphasis on future prospects in early diagnosis, biomarker integration, and tumor response evaluation.
